# TV Time but Not Computer Time Is Associated with Cardiometabolic Risk in Dutch Young Adults

**DOI:** 10.1371/journal.pone.0057749

**Published:** 2013-02-27

**Authors:** Teatske M. Altenburg, Marlou L. A. de Kroon, Carry M. Renders, Remy HiraSing, Mai J. M. Chinapaw

**Affiliations:** 1 Department of Public and Occupational Health, VU University Medical Center, EMGO Institute for Health and Care Research, Amsterdam, The Netherlands,; 2 Department of Health Sciences, VU University, EMGO Institute for Health and Care Research, Amsterdam, The Netherlands; Tulane School of Public Health and Tropical Medicine, United States of America

## Abstract

**Background:**

TV time and total sedentary time have been positively related to biomarkers of cardiometabolic risk in adults. We aim to examine the association of TV time and computer time separately with cardiometabolic biomarkers in young adults. Additionally, the mediating role of waist circumference (WC) is studied.

**Methods and Findings:**

Data of 634 Dutch young adults (18–28 years; 39% male) were used. Cardiometabolic biomarkers included indicators of overweight, blood pressure, blood levels of fasting plasma insulin, cholesterol, glucose, triglycerides and a clustered cardiometabolic risk score. Linear regression analyses were used to assess the cross-sectional association of self-reported TV and computer time with cardiometabolic biomarkers, adjusting for demographic and lifestyle factors. Mediation by WC was checked using the product-of-coefficient method.

TV time was significantly associated with triglycerides (B = 0.004; CI = [0.001;0.05]) and insulin (B = 0.10; CI = [0.01;0.20]). Computer time was not significantly associated with any of the cardiometabolic biomarkers. We found no evidence for WC to mediate the association of TV time or computer time with cardiometabolic biomarkers.

**Conclusions:**

We found a significantly positive association of TV time with cardiometabolic biomarkers. In addition, we found no evidence for WC as a mediator of this association. Our findings suggest a need to distinguish between TV time and computer time within future guidelines for screen time.

## Introduction

Sedentary behaviour, especially TV viewing, has been indicated as an important lifestyle risk factor of type 2 diabetes and CVD, independently of physical activity [1]–[3]. Recent reviews have examined the association of TV viewing only [4], [5] and broad measures of sedentary behaviour, such as screen time and total sedentary time [6]–[8], with health outcomes in adults. A review including predominantly cross-sectional studies, found evidence on a positive association of TV viewing with weight status and metabolic syndrome, while mixed results were found for an association with blood lipids, blood pressure and type 2 diabetes [5]. A recent meta-analysis on prospective cohort studies concluded that the risk of type 2 diabetes and CVD linearly increased with the number of hours per day of TV viewing [4]. However, insufficient evidence was found for a longitudinal relationship of sedentary time with weight gain, body weight/BMI and the risk for overweight and obesity [4], [6], [8] and contradictory results were found for a longitudinal relationship with cardiometabolic biomarkers [4]–[6], [8].

Up to now, only two studies in adults examined the association of other sedentary activities than TV viewing with risk of obesity and type 2 diabetes mellitus [9], [10]. In the study of Hu et al [9] among US women (aged 30–55 years), TV viewing, sitting at work and passive transport were significantly positively associated with increased risk of obesity and type 2 diabetes. Other sedentary behaviours, such as sitting while eating and reading, were significantly positively associated with increased risk of type 2 diabetes, but not obesity [9]. Pinto Pereira et al [10] found among British adults (aged 44–45 years) that higher TV time was associated with an adverse profile for cardiometabolic biomarkers (e.g. blood pressure, triglycerides, total cholesterol). In contrast, higher work sitting time was not associated to cardiometabolic biomarkers in women, and only weakly associated with cardiometablic biomarkers in men [10].

TV viewing appeared to be a suitable marker for sedentary behaviour in women but not in men [11]. This may be due to gender differences in energy intake and energy expenditure during sedentary activities. Different sedentary activities such as TV viewing and computer use might have different health effects. In a study in adults and children, an increase in energy expenditure during seated video game play was found compared to rest, indicating that video gaming is not an entirely passive activity, even when played seated [12]. In addition, experimental studies have demonstrated that TV viewing is related to increased consumption of energy dense snacks and beverages [13]–[15]. Video gaming and computer use may therefore have smaller associations with cardiometabolic biomarkers than TV viewing. To the best of our knowledge, no studies have examined whether screen behaviours other than TV viewing, such as using the computer (e.g. gaming, chatting), are associated with cardiometabolic biomarkers in adults.

**Figure 1:**
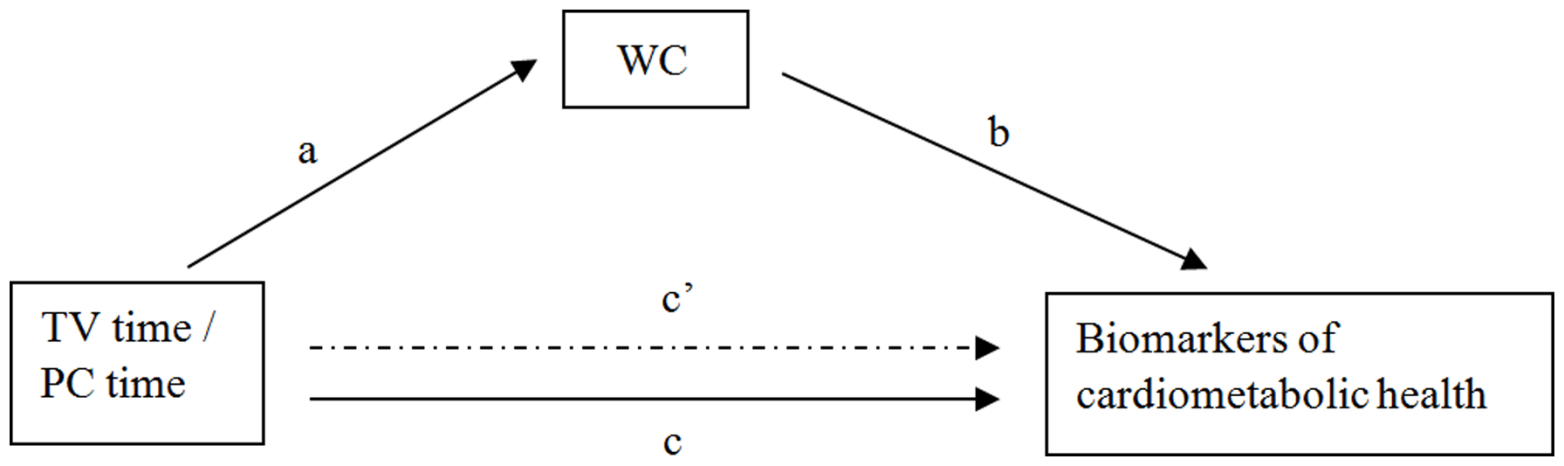
Conceptual model for the mediated effect of WC on the association of TV time and computer (PC) time with biomarkers of cardiometabolic risk. a = Association between TV time and PC time with WC. b = Association between WC and biomarkers of cardiometabolic risk. c = Total association of TV time and PC time with biomarkers of cardiometabolic risk, unadjusted for WC. c′ = Direct association of TV time and PC time with biomarkers of cardiometabolic risk, adjusted for WC.

We aimed to examine the cross-sectional association of TV time and computer time with cardiometabolic biomarkers in young adults (aged 18–28 years). We hypothesize that TV time has a stronger detrimental association with cardiometabolic biomarkers than computer time. A potential explanatory mechanism of this association in young adults may be that excessive screen time results in increased abdominal adiposity, which in turn may lead to in higher cardiometabolic risk. Therefore, our second aim was to examine the mediating effects of waist circumference (WC) on this association (see Figure 1).

## Methods

### Ethics Statement

This study was approved by the Medical Ethics Committee of the VU University Medical Center in Amsterdam and was in accordance with the Declaration of Helsinki. All participants gave their written informed consent prior to their inclusion in the study.

### Design and participants

The young adults selected for this study were participants in the follow-up study of the Terneuzen Birth Cohort. The Terneuzen Birth Cohort was initiated to evaluate and monitor the initiation and duration of breastfeeding, and consists of all individuals who were born between 1977 and 1986 in the city of Terneuzen (n = 2604). Data on height and weight from birth to adolescence could be retrieved from the child health care files for 1701 subjects aged 18–28 years.

A follow up measurement was performed in 2004–2005 and included physical examination, blood tests and questionnaires to collect data on lifestyle factors, such as sedentary behaviour, cigarette smoking and dietary behaviour, and socio-demographics. Of the initial 1701 children, 642 young adults participated in the follow up measurement. The males and females in this follow-up measurement were comparable to the original cohort regarding baseline characteristics, e.g. age, birth weight, BMI SDS at birth. The only significant difference was gender (41% males vs. 51% in the original cohort, p<0.05). For further details see de Kroon et al [16].

For the present analysis, participants with self-reported hypertension, diabetes and dyslipidaemia were excluded. In total, data of 634 participants were included in the present analysis. Due to missing data, sample sizes varied between the different analyses (see Table 1 for the sample size for each variable).

**Table 1:** Participant characteristics (Mean±SD).

		n	All (n = 634)	Male (n = 246)	Female (n = 388)
Age, years		634	23.0±2.9	23.2±3.0	22.9±2.9
Overweight# (obese), %		634	21.9 (3.6)	22.0 (2.8)	21.9 (4.1)
Cardiometabolic biomarkers					
BMI, kg/m2		634	23.1±3.5	22.9±3.3	23.3±3.6
WC, cm		633	80.5±10.1	84.0±9.4*	78.3±9.8
SSF, mm		627	57.6±25.2	44.6±20.9*	65.9±24.3
SBP, mmHg		631	126.2±13.4	134.3±12.1*	121.1±11.6
DBP, mmHg		631	75.7±8.5	75.5±8.1	75.8±8.7
Total cholesterol, mmol/l		564	4.5±0.9	4.4±0.9*	4.6±0.9
HDL-C, mmol/l		564	1.4±0.3	1.2±0.2*	1.5±0.3
Triglycerides, mmol/l		564	0.9±0.5	0.9±0.6	0.9±0.5
Glucose, mmol/l		562	5.1±0.5	5.2±0.5*	5.0±0.5
Insulin, mmol/l		235	12.8±7.9	10.7±4.7*	13.9±8.8
HbA1c, mmol/l		423	5.1±0.8	5.1±0.3	5.2±1.0
hsCRP, mmol/l		555	2.6±5.0	1.3±3.2*	3.5±5.8
MetS, %		559	6.8	7.5	6.3
zCM-risk score		634	−0.01±0.6	0.3±0.5*	−0.2±0.6
Screen time					
TV viewing, min/day		627	148.7±127.1*	132.7±117.7	158.7±131.9
Computer use, min/day		615	151.2±170.0*	188.2±182.6	127.3±157.1
Cigarette smoking1, %		444	50	52	48
Covariates					
Alcohol use2, %		631			
	never		30	18*	37
	moderate (often)		62 (8)	66 (15)	60 (3)
Breakfast, days/week		630	5.7±2.1	5.2±2.4*	6.0±1.9
BMI mother, kg/m2		543	25.3±4.2	24.9±3.8	25.6±4.4

# Indicates overweight only; ^*^Indicates significant gender difference based on student's T-test for unpaired samples (continuous variables) or chi-square (χ^2^) test (categorical variables).

1 Having smoked a cigarette in the last week; ^2^Alcohol usage (never, moderate, often).

BMI = body mass index; WC = waist circumference; SSF = skinfold thickness; SBP = systolic blood pressure; DBP = diastolic blood pressure; HDL-C = high density lipoprotein cholesterol; HbA1c = glycated Haemoglobin; hsCRP = high-sensitivity C-reactive protein; MetS = metabolic syndrome; zCM-risk score = sumscore for clustered cardiometabolic risk.

### Measurements

#### Cardiometabolic biomarkers

Cardiometabolic biomarkers included indicators of overweight, blood pressure, blood levels of fasting plasma glucose, insulin glycated haemoglobin (HbA1c), cholesterol, high density lipoprotein cholesterol (HDL-C), triglycerides and high-sensitivity C-reactive protein (hsCRP).

Fasting venous blood samples were drawn in the clinical chemistry laboratory of the Community Hospital in Terneuzen, by two trained assistants according to a standardised protocol. After centrifugation (10 minutes 1500xG), plasma levels of glucose, insulin, HbA1c, total cholesterol, HDL-C, triglyceride and hsCRP were analysed using a routine clinical chemical analyse (Synchron LX20PRO).

Clustering of cardiometabolic biomarkers, known as the metabolic syndrome is an important predictor in the development of CVD [17]–[19]. Therefore, we calculated a standardised clustered cardiometabolic risk score from fasting glucose, insulin, HDL-C, TG, WC and blood pressure (SBP and DBP averaged). Each individual biomarker was first converted to z-scores. Z-scores of individual biomarkers were averaged to construct the clustered cardiometabolic risk score with higher values indicating higher risk ([20], [21]). In addition, for descriptive purposes, we defined the metabolic syndrome using the most widely accepted NCEP Adult Treatment Panel III definition, which applies if at least three out of the following five components are met [17]: increased WC (>102 cm for men or >88 cm for women), elevated triglycerides (≥1.7 mmol/l), reduced HDL-C (<1.0 mmol/l for men or <1.3 mmol/l for women), increased blood pressure (systolic blood pressure ≥130 mmHg or diastolic blood pressure ≥85 mmHg) and elevated fasting plasma glucose (≥5.6 mmol/l).

Body mass index (BMI), WC and skinfold thickness (SSF) (at the triceps, biceps, subscapular and suprailiac sites) were included as measures of overweight. All measurements were performed by two trained assistants using a standardised protocol with participants dressed in underwear. Body height (m) was measured to the nearest 0.1 cm using a portable stadiometer. Body weight (kg) was measured to the nearest 0.1 kg using an electronic self-zeroing scale. Body height and body weight were measured to calculate BMI (kg/m^2^). For descriptive purposes only, participants were categorised based on their BMI as having a healthy weight (BMI<25), being overweight (25≤BMI<30) or being obese (BMI≥30). WC (cm) was measured to the nearest 0.5 cm using a flexible band. Skinfold thickness (mm) was measured to the nearest 0.2 mm using a Holtain skinfold calliper, and by averaging 3 measurements.

Blood pressure was measured on the left upper arm using the fully automatic Omron 5-1, and averaging 2 measurements for diastolic blood pressure (DBP) and systolic blood pressure (SBP) (with a 5 min rest interval).

#### Screen time

Screen time, defined as hours per week, was assessed by self-report. Participants were asked how many days per week (in separate questions for weekdays and weekend days) and how many hours per day they spent on average on 1) working or chatting on the computer, 2) playing video games on the computer, X-box or PlayStation, and 3) viewing TV or video. The average time spent using the computer was calculated by summing the time spent on working, chatting and playing video games.

#### Covariates

The following lifestyle and socioeconomic covariates of the young adults were considered as confounders in the present study: gender, age, birth weight, cigarette smoking, alcohol use, breakfast frequency, moderate-to-vigorous physical activity (MVPA), the presence of CVD in the family, education level and BMI of the mother.

Except for birth weight, which was obtained from the child health care files of the Terneuzen Birth Cohort, all covariates were assessed by self-report at follow up. Cigarette smoking was defined as having smoked a cigarette in the past week or not. Alcohol use was classified as drinking 1) never, 2) regularly (defined as drinking up to 7 glasses per week) or 3) often (defined as drinking more than 7 glasses per week). Breakfast frequency was defined as the frequency of taking breakfast per week. MVPA was assessed using the validated SQUASH (Short QUestionnaire to ASsess Health-enhancing physical activity) questionnaire [22]. MVPA was calculated by summing time spent in active transport, physical activity at work or school and leisure time physical activity and sports (min/day). The presence of CVD in the family was defined as having a parent, brother or sister diagnosed with CVD or not. BMI of the mother was defined as weight/height^2^. Education level was categorised as low, medium or high.

Gender, cigarette smoking, alcohol use and breakfast behaviour of the young adults and BMI of the mother confounded the association of TV time and computer time with cardiometabolic biomarkers and were therefore included in the present analysis.

### Statistics

Participant characteristics were summarised by means, standard deviations (SD) or percentages. Gender differences were examined using chi-square (χ^2^) tests for categorical data and Student's unpaired *t*-tests for continuous data.

Linear regression analysis was used to assess the association of TV time and computer time with individual cardiometabolic biomarkers and the clustered cardiometabolic risk score. Adjustments were made for gender, cigarette smoking, alcohol use, breakfast frequency and maternal BMI (model 1). In a subsequent model we additionally adjusted for computer time in the association with TV time, and for TV time in the association with computer time (model 2). The correlation between TV time and computer time was 0.06 (P = 0.12).

To check for mediation by WC the product-of-coefficient method (a*b) of MacKinnon was applied [23]. Using this method a series of regression analyses was performed. All regression analyses were adjusted for demographic and lifestyle factors, and computer time in the association with TV time, and TV time in the association with computer time. First, we assessed the direct association of TV time and computer time with cardiometabolic biomarkers (main effect, c-path). Second, we assessed the association of TV time and computer time with WC (a-path). Third, we assessed the association between WC and cardiometabolic biomarkers, adjusted for TV time and computer time (b-path). The mediating effect is the product of the a and b paths (a*b) and provides an estimate of the magnitude of the mediation effect in the units of the outcome variable. The statistical significance of a mediating effect was tested by dividing the products of the coefficients a and b by its standard error (SEab = √(a2*SEb^2^+b2*SEa^2^)). The product of coefficient method suggests that potential mediating effects should also be analysed if the c-path (main effect) is not significant [23].

Mediation effects of WC were not examined for the associations in which other indicators of overweight (BMI, skinfolds) and the clustered cardiometabolic risk score were modelled as outcome variables. All statistic procedures were performed using SPSS software (version 17.0). Statistical significance was set at P<0.05.

## Results

### Participant characteristics

Table 1 shows the participant characteristics for men and women separately. Males spent significantly less time watching TV and more time using the computer than females. WC, SBP, skinfold thickness, total cholesterol, HDL-C, insulin, glucose, hsCRP and the clustered cardiometabolic risk score were significantly higher for males than for females. Males reported a higher alcohol use and lower breakfast frequency than females.

### TV viewing time and cardiometabolic risk

Table 2 shows the associations between TV time and cardiometabolic biomarkers. After adjustment for demographic and lifestyle factors, TV time was significantly positively associated with BMI (B = 0.03; [CI = 0.01; 0.05]), SSF (B = 0.02; [CI = 0.002; 0.04]), WC (B = 0.09; [CI = 0.02; 0.16]) and plasma levels of triglycerides (B = 0.05; [CI = 0.001; 0.01]) and insulin (B = 0.13; [CI = 0.04; 0.22]) (model 1). After additional adjustment for computer time, the association of TV time with triglycerides (B = 0.004; [CI = 0.0004; 0.01]) and insulin (B = 0.10; [CI = 0.01; 0.20]) remained significant (model 2). In addition, TV time was significantly positively associated with the clustered cardiometabolic risk score (B = 0.01; [CI = 0.001; 0.01]), after adjustment for demographic and lifestyle factors (model 1). This association was no longer significant after additional adjustment for computer time (model 2).

**Table 2:** Cross-sectional associations (unstandardised regression coefficients (b) [95% CI]) of TV time and computer time with cardiometabolic biomarkers in Dutch young adults.

	TV time (hrs/week)		Computer time (hrs/week)	
	**Model 1**	**Model 2**	**Model 1**	**Model 2**
BMI, kg/m^2^	**0.03 [0.01; 0.05]**	0.01 [−0.01; 0.036]	0.000 [−0.02; 0.02]	0.003 [−0.02; 0.02]
SSF, mm	**0.02 [0.002; 0.04]**	0.02 [−0.00; 0.04]	0.10 [−0.03; 0.22]	0.10 [−0.02; 0.23]
WC, cm	**0.09 [0.02; 0.16]**	0.05 [−0.02; 0.12]	−0.03 [−0.08; 0.03]	−0.02 [−0.07; 0.03]
SBP, mmHg)	0.001 [−0.08; 0.08]	−0.01 [−0.09; 0.08]	−0.01 [0.08; 0.05]	0.003 [−0.06; 0.07]
DBP, mmHg	0.02 [−0.04; 0.07]	−0.003 [−0.06; 0.06]	0.03 [−0.01; 0.08]	0.04 [−0.004; 0.09]
CHOL, mmol/l	0.01 [−0.000; 0.01]	0.01 [−0.001; 0.01]	0.002 [−0.003; 0.007]	0.002 [−0.003; 0.01]
HDL-C, mmol/l	−0.002 [−0.004; 0.0002]	−0.001 [−0.003; 0.001]	0.000 [−0.001; 0.002]	0.000 [−0.001; 0.002]
TRI, mmol/l #	**0.005 [0.001; 0.01]**	**0.004 [0.000; 0.01]**	0.001 [−0.002; 0.004]	0.001 [−0.002; 0.005]
Glucose, mmol/l	0.000 [−0.003; 0.004]	−0.002 [−0.005; 0.002]	0.002 [−0.001; 0.004]	0.002 [−0.001; 0.005]
Insulin, mmol/l	**0.13 [0.04; 0.22]**	**0.10 [0.01; 0.20]**	0.03 [−0.05; 0.10]	0.02 [−0.05; 0.10]
HbA1c, mmol/l	−0.003 [−0.01; 0.01]	−0.003 [−0.01; 0.01]	−0.003 [−0.01; 0.004]	−0.003 [−0.01; 0.004]
hsCRP, mmol/l	0.03 [−0.01; 0.06]	0.01 [−0.03; 0.05]	−0.01 [−0.04; 0.02]	−0.01 [−0.04; 0.03]
zCM-risk score	**0.01 [0.001; 0.01]**	0.003 [−0.001; 0.01]	0.000 [−0.003; 0.003]	0.001 [−0.002; 0.003]

Model 1: Adjusted for gender, cigarette smoking, alcohol use, breakfast frequency and maternal BMI; Model 2: Additionally adjusted for computer time in the associations with TV time and TV time in the associations with computer time.

Bold – significant associations (P<0.05).

BMI = body mass index; WC = waist circumference; SSF = skinfold thickness; SBP = systolic blood pressure; DBP = diastolic blood pressure; CHOL =  total cholesterol; HDL-C = high density lipoprotein cholesterol; TRI =  triglycerides; HbA1c = glycated hemoglobin; hsCRP = high-sensitivity C-reactive protein; zCM-risk score = sumscore for clustered cardiometabolic risk.

### Computer time and cardiometabolic risk

Computer time was not significantly associated with any of the cardiometabolic biomarkers, or the clustered cardiometabolic risk score (Table 2).

### Mediation of WC

Table 3 shows the mediation effects of WC. We found no evidence for a mediating effect of WC in the association of TV time or computer time with cardiometabolic biomarkers.

**Table 3:** Mediation effect (estimate [95% CI]) of WC on the association of TV time and computer time with cardiometabolic biomarkers.

	Association between TV time/computer time and mediator (path a)	Association between mediator and cardiometabolic biomarkers (path b)	Mediation effect in the association with TV time (a * b)	Mediation effect in the association with computer time (a * b)
WC, cm				
TV time (hrs/week)	0.05 [−0.02; 0.12]			
Computer time (hrs/week)	−0.02 [−0.07; 0.03]			
SBP, mmHg		0.27 [0.15; 0.40]	0.02 [−0.01; 0.04]	−0.01 [−0.02; 0.01]
DBP, mmHg		0.14 [0.04; 0.23]	0.01 [−0.003; 0.02]	−0.003 [−0.01; 0.01]
CHOL, mmol/l		0.03 [0.02; 0.04]	0.001 [−0.005; 0.003]	−0.001 [−0.002; 0.001]
HDL-C, mmol/l		−0.003 [−0.01; 0.000]	−0.0002 [−0.0005; 0.0001]	0.0001 [−0.0001; 0.0003]
TRI, mmol/l		0.02 [0.02; 0.03]	0.001 [−0.0004; 0.003]	−0.0004 [−0.0002; 0.001]
Glucose, mmol/l		0.002 [−0.004; 0.01]	0.0001 [−0.0002; 0.0004	0.0000 [−0.0002; 0.0001]
Insulin, mmol/l		0.25 [0.12; 0.38]	0.01 [−0.01; 0.03]	−0.01 [−0.02; 0.01]
HbA1c, mmol/l		0.001 [−0.01; 0.02]	0.0001 [−0.001; 0.001]	0.0000 [−0.0004; 0.0003]
hsCRP, mmol/l		0.09 [0.03; 0.15]	0.01 [−0.002; 0.01]	−0.002 [−0.01; 0.003]

All associations were adjusted gender, cigarette smoking, alcohol use, breakfast behaviour, maternal BMI, and for computer use in the association with TV time, and for TV time in the association with computer time.

WC = waist circumference; SBP = systolic blood pressure; DBP = diastolic blood pressure; CHOL = total cholesterol; HDL-C = high density lipoprotein cholesterol; TRI = triglycerides; HbA1c = glycated hemoglobin; hsCRP = high-sensitivity C-reactive protein.

## Discussion

This study aimed to examine the association between TV time and computer time respectively, and cardiometabolic biomarkers in Dutch young adults. To the best of our knowledge, our study is the first examining the association of TV time and computer time separately with cardiometabolic biomarkers in young adults. We found that TV time was significantly associated with blood levels of triglycerides and insulin, after adjustment for demographic and lifestyle factors and computer time. In addition, we found no evidence for WC to mediate these associations.

Our finding that TV time was associated with individual biomarkers of cardiometabolic health is in line with previous reviews on TV time in adults [4], [5]. Our findings indicate that, in relatively young and healthy adults, watching 1 additional hour TV per day (i.e. 7 hrs/week) was associated with 0.03 mmol/l (3%) higher triglyceride levels and 0.7 mmol/l (5.5%) higher insulin levels. Although these associations may seem small, it may have considerable health risks when sustained over long periods of time. Long-term prospective research is required to establish the clinical relevance of these associations. We found no association between TV time and the clustered cardiometabolic risk score, which is in contrast to previous studies using a similar cardiometabolic risk score [24], [25]. The relatively healthy population in our study (i.e. 7% metabolic syndrome in the present study, compared to 11 and 20% in previous studies [24], [25]), may have masked an association between TV time and the clustered cardiometabolic risk score.

We found a significant association of TV time with triglycerides but not with total cholesterol and HDL-C. This is in line with previous cross-sectional studies, that found a stronger association of TV time with triglycerides than with cholesterol [5]. Similarly, we found a significant association of TV time with insulin but not with glucose. This is in line with a previous study examining the association of sedentary time and cardiometabolic biomarkers [26].

In agreement with recent studies in children [27], [28], we found no association of computer time with individual cardiometabolic biomarkers or the clustered cardiometabolic risk score. An explanation for the different associations for TV time and computer time could be a difference in energy intake during TV viewing and computer use. A recent review of studies on the association between TV viewing and diet concluded that TV viewing is associated with a less healthy diet (e.g. reduced fruit and vegetable consumption, increased intake of energy-dense snacks and drinks) [29]. Future studies need to examine whether computer use is associated with unhealthy dietary habits as well. A second explanation could be a difference in energy expenditure. Using the computer may lead to a higher energy expenditure than TV viewing [12] since computer use may require higher muscle activity. Moreover, our questionnaire did not distinguish between sedentary and active computer games. A third explanation might be residual confounding. For example, computer time includes both work and leisure computer time, whereas TV time only includes watching TV during leisure time. Residual confounding could be factors related to employment or study that we did not take into account.

In order to examine whether concurrent energy surplus may explain the adverse health effects of limited muscle activity, a recent study examined the acute effects of sitting, with and without energy surplus, on insulin action. It was found that one day of sitting considerably reduced insulin action [30]. This effect was minimised, but not prevented, when energy intake was reduced to match energy expenditure. This suggests that the different associations for TV time and computer time might be due to differences in muscle activity. Experimental studies have demonstrated that a lack of muscle activity leads to suppression of skeletal muscle lipoprotein lipase (LPL) activity [31], [32]. Computer time might be less passive and thus lead to a smaller suppression of skeletal LPL activity than TV viewing, resulting in lower cardiometabolic risk.

Although previous studies have adjusted for WC in the analysis [24], [25], only one study examined WC as a mediator applying a complete mediation analysis [33]. Stamatakis & Hamer [33] found that the association of TV time with blood pressure, total cholesterol and HDL-C cholesterol was partly mediated by BMI or WC in adults aged 16–65 years. In contrast, we found no evidence that WC mediated the association of TV time or computer time with cardiometabolic biomarkers. A possible explanation for these contrasting results may be the relatively young and healthy population, and the small variability in WC in our study compared to the study of Stamatakis & Hamer [33]. Our results suggest that abdominal adiposity cannot explain the adverse association between TV time and cardiometabolic health. Future studies are needed to confirm this finding and to examine the underlying biological mechanisms.

One of the strengths of the present study is the distinction between TV viewing and computer use. Additional strengths include the objectively assessed cardiometabolic biomarkers, the large variety of confounders that were included in the analyses and the mediation analysis of WC. A limitation of the present study is the cross-sectional design. The simultaneous measurement of predictors, outcomes and hypothetical mediator, implicates that no conclusions about causality can be drawn. Second, data on TV time, computer time and a number of covariates (cigarette smoking, alcohol use, breakfast frequency, MVPA, familiar CVD and BMI of mother) relied on self-report, which is sensitive to recall bias and socially desirable answers. The reliability and validity of these self-reported measures were unknown, which further limits our study. An additional limitation is the use of breakfast frequency as a proxy for dietary habits. Fourth, self-reported measures of computer time included both work related and leisure time computer use and might therefore be less accurately recalled than TV time. In addition, we could not distinguish using the computer for TV viewing. Fifth, as in most cohorts, there was a loss to follow-up. However, selection bias is unlikely, since gender was the only significant difference in baseline characteristics between this follow-up study and the original cohort. Moreover, there is no reason to assume that the association of TV time and computer time with cardiometabolic biomarkers differs between males and females. Another limitation of our study is the number of statistical tests performed, which implies that the results should be carefully interpreted.

We conclude that TV time was positively associated with cardiometabolic biomarkers. We found no evidence that abdominal adiposity mediated the association between TV time and cardiometabolic biomarkers.

Our findings suggest that different types of screen behaviours may have different effects on health outcomes. Although prospective evidence is needed to confirm a causal relationship, our findings suggest that TV time and computer time should be considered as separated classes of screen behaviour. Future guidelines for screen behaviour may therefore need to distinguish between time spent watching TV and time spent using the computer.
